# Physiologic and Symptomatic Responses to Low-Level Substances in Individuals with and without Chemical Sensitivities: A Randomized Controlled Blinded Pilot Booth Study

**DOI:** 10.1289/ehp.7198

**Published:** 2005-05-19

**Authors:** Michel R. Joffres, Tara Sampalli, Roy A. Fox

**Affiliations:** Nova Scotia Environmental Health Centre, Fall River, Nova Scotia, Canada

**Keywords:** complex mixtures, electrodermal response, multiple chemical sensitivity, pilot project, randomized controlled trial, skin conductance

## Abstract

We conducted a pilot study using a randomized, single-blind, placebo-controlled exposure among 10 individuals with and 7 without reported chemical sensitivities in a dedicated testing chamber. Objectives of the study were to explore the length of the adaptation period to obtain stable readings, evaluate responses to different substances, and measure the level and type of symptomatic and physiologic reactions to low-level exposures. Reported and observed symptoms, electrodermal response, heart rate, skin temperature, surface electromyogram, respiratory rate, contrast sensitivity, and the Brown-Peterson cognitive test were used and compared between cases and controls and between test substances (glue, body wash solution, dryer sheet) and control substances (unscented shampoo and clean air). Subjects with chemical sensitivities (cases) took longer to adapt to baseline protocols than did controls. After adaptation, despite small study numbers, cases displayed statistically significant responses (all measures, *p* < 0.02) in tonic electrodermal response to test substances compared with controls and compared with the control substance. Symptoms were also higher in cases than in controls for the body wash solution (*p* = 0.05) and dryer sheets (*p* = 0.02). Test–retest showed good agreement for both symptoms and tonic electrodermal responses (McNemar’s test, *p* = 0.32 and *p* = 0.33, respectively). Outside of skin conductance, other measures had no consistent patterns between test and control substances and between cases and controls. This study shows the importance of using an adaptation period in testing individuals with reported chemical sensitivities and, despite small numbers, raises questions about underlying mechanisms and level of reactivity to low-level chemical exposures in sensitive individuals.

Despite the controversial nature of multiple chemical sensitivities (MCS)/environmental sensitivities (ES), the following consensus criteria (Bartha et al. 1999) have been proposed to define MCS/ES: The symptoms are reproducible with repeated chemical exposure; the condition is chronic; low levels of exposure (lower than previously tolerated) result in manifestations of the syndrome; the symptoms improve or resolve when the incitants are removed; responses occur to multiple chemically unrelated substances; and symptoms involve multiple organ systems. These criteria are based upon previous definitions (Cullen 1987; Nethercott et al. 1993; Randolph 1961).

Some researchers consider another term, “idiopathic environmental intolerances” (IEI), more appropriate [International Programme on Chemical Safety (IPCS) 1996] and believe it should be restricted to individuals with absolutely no abnormalities except a self-reported abhorrence to chemicals. Many people with chemical sensitivities have other conditions and symptoms such as asthma, rhinosinusitis, dermatitis, and headaches triggered by chemical exposures and would not meet this restrictive definition of idiopathic environmental intolerances. Our previous study (Joffres et al. 2001) identified a common set of consistent symptoms that follow exposure: difficulty in concentrating, fatigue, forgetfulness, irritability, itchy or burning eyes, sneezing, and hoarseness or loss of voice. The prevalence of ES is not negligible (Kreutzer et al. 1999; Meggs et al. 1996), and physicians, even those most familiar with these conditions, often overlook such a syndrome (Kutsogiannis and Davidoff 2001). Questions remain unanswered regarding the etiology, associated mechanisms, and inconsistency in pattern of symptoms. Several reports (Fiedler and Kipen 1997; Kipen and Fiedler 2002a, 2002b; Sparks et al. 1994) have outlined some of the different viewpoints on etiology and physiopathology. Meggs (1995) and Bascom et al. (1997) have proposed a neurogenic inflammation model. Sensitization or kindling of olfactory-limbic pathways by acute or chronic exposure has also been proposed (Bell et al. 1997, 1999; Miller 2001). Researchers for the IPCS (1996) proposed that

the key experiment is to determine in a double-blind challenge study whether subjects with “IEI” successfully discriminate between exposures to environmental factors (including to which illness is attributed) and placebo. If the subjective response (appearance of symptoms) of test subjects is able to discriminate between exposure to test chemicals and placebos, in a blinded design, this would suggest the operation of a toxicological mechanism in which culpable agents interact with tissue targets to trigger a receptor-mediated pathophysiological response.

A few studies have attempted to look at experimental testing of affected individuals (Fiedler et al. 2000; Fiedler and Kipen 2001; Rea et al. 1991; Staudenmayer et al. 1993). A critical factor that may have been overlooked in some of these studies is the adaptation of subjects to baseline study protocols. Adaptation is defined here as the period taken by the subjects in a study to get used to the general study protocols, obtaining stable physiologic (i.e., skin conductance and symptomatic) readings before the actual introduction of substances. We realized that with our patient population expressing a high level of reactivity, we would need to get stable readings before introducing any test substance. Otherwise, we might obtain erratic physiologic or symptomatic responses because of testing conditions, which would make it impossible to differentiate challenge substances from placebos. Surprisingly, we have not found any psychophysiologic literature discussing the importance of stabilizing readings before starting experiments other than the usual caution of a few minutes of relaxation. Most studies have either used a set period for adaptation (Fiedler et al. 2000) or not considered this factor at all (Staudenmayer et al. 1993).

Therefore, we hypothesized that individuals with ES may require more time to adapt to the experimental conditions compared with controls without chemical sensitivities. Also, we hypothesized that each individual may have a different adaptation period. We also wanted to explore the type of measure that would be the most useful to detect change and therefore included several psychophysiologic measures.

Addressing the issue of symptom development has also been critical to research on these illnesses. There is a need to see how symptoms develop and what triggers are associated with symptoms in individuals with ES. Is there a relation between symptoms and skin conductance response during exposure to chemicals? Estimating the level of reactivity of cases and controls to the substances was another major objective of this pilot study.

Hence, to answer these questions, we conducted a pilot, blinded, controlled booth study at the Nova Scotia Environmental Health Centre, a government-funded facility dedicated to research and management of individuals with ES.

## Materials and Methods

### Inclusion criteria.

Subjects with sensitivities were selected from the last 50 new patients enrolled at the Nova Scotia Environmental Health Centre who fulfilled the consensus criteria (Bartha et al. 1999) and who gave informed consent to participate in the study. The Dalhousie University Health Sciences Human Research Ethics Board approved the study protocol. Controls, without known chemical sensitivities, were recruited from friends of the patients and from advertisement in local churches.

### Exclusion criteria.

Subjects were excluded from the study if they had any other major illnesses such as cancer (outside of skin cancer or past cancer without relapse in the preceding 5 years), insulin-dependent diabetes, stage 2 or 3 hypertension (systolic/diastolic blood pressure: stage 2, 160–179/100–109; stage 3, > 180/> 110), history of myocardial infarction, angina pectoris, stroke, or psychiatric disorders such as major depressive disorder, schizophrenia, shared psychotic disorder, dementia, or drug dependence.

A total of 12 cases (all women; mean age, 40 years; range, 25–60 years) and 7 controls (all women; mean age, 42 years; range, 26–59 years) gave an informed consent to participate and fulfilled the initial inclusion/exclusion criteria of the study. The subjects were matched within age, education, and ethnicity categories.

### Challenge booth.

**Figure 1:**
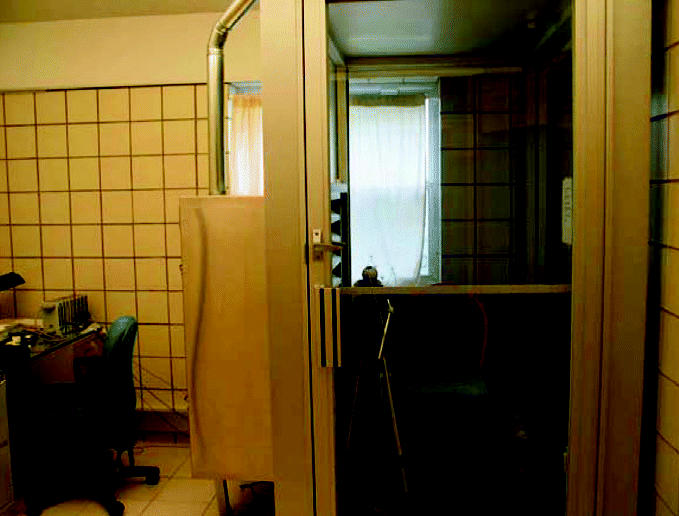
Challenge booth and testing conditions.

The study was conducted in a dedicated room that included the challenge booth (Figure 1) made from inert materials with no apparent odors, allowing introduction of materials that release various chemicals into the environment. The booth is a glass room with steel framing with dimensions of 2.2 m (7.2 ft) in height by 1.2 m (4 ft) in width by 1.8 m (6 ft) in depth. The construction materials and procedure used have received careful consideration toward making it suitable for research work on individuals with chemical sensitivities. The air-flow, temperature, and lighting in the facility can be varied to increase the comfort level of the subjects. A side box attached to the booth was used to introduce chemicals into the air stream. The door to the side box opens on one side of the booth so the subjects are unaware of the substance being introduced into the booth. Air is allowed to enter the side box into an inlet provided on the frosted side of the booth. The inlet height is at the breathing level of the occupant. The air entering the booth is 100% outdoor air that has been filtered before being distributed throughout the clinic, not recirculated, and is then directly vented to the outdoors through an exhaust located in the ceiling of the booth.

### Test substances.

Test substances for the study were those commonly reported by affected people to cause reactions: common glue, a scented body wash solution, dryer sheet, and for control substances, unscented shampoo and clean air. These substances were contained in a closed metal box, introduced through the side box, and passively released in the booth airflow. A new dryer sheet was used for each session. Patients could not smell the substance because of nose plugs.

### Outcome measures.

Before and after each booth session, subjects answered a symptom questionnaire on symptoms reported by Joffres et al. (2001; e.g., eye irritation, throat irritation, sleepiness, headache) as the top symptoms experienced by our patient population after an exposure. The questionnaire measured irritation on an ordinal scale of 0–10, with 0 being barely detectable and 10 being strongest detectable (Joffres et al. 2001). The booth environment (light, sound, and temperature) was also rated before and after each session on a scale of 0–10 (0 = poor, 10 = excellent). The purpose of the adaptation sessions was to reduce the number and severity of symptoms reported to the nurse among reactive subjects. The adaptation sessions identified symptomatic responses shown by subjects to the baseline protocols of the study such as wearing nose plugs, wearing respiratory belt, and the other BIOPAC (BIOPAC Systems Inc., Goleta, CA, USA) measures.

During the testing period, we compared pre- and postsymptom scores from that day, and coded any change in score or type of symptom as a positive response. We disregarded the symptoms that occurred consistently during the adaptation sessions (baseline symptomatic responses) while computing a response toward challenge substances.

After the booth session, subjects recorded and reported to the nurse any changes they observed during the next 8-hr period. They were instructed not to visit malls or other similar places where they may be exposed to other substances 8 hr before and 8 hr after each booth session. Because many of our patients reported effects lasting up to 4 or 5 days after a booth session, subjects were exposed to only one substance at a time and had a minimum of 1 week between two booth sessions.

We used the Brown-Peterson test (Peterson and Peterson 1962) to test a variation in the short-term memory span of the subjects after a booth exposure. Subjects were given a series of trigrams of letters; each trigram was followed by a number countdown challenge, after which subjects were asked to recall the trigram. Short-term memory for 9, 18, and 36 sec intervals was examined. The test was conducted before and after each exposure session.

Contrast sensitivity (Schreiber et al. 2002), which provides a detailed assessment of spatial vision and is sometimes recommended as a test to screen visual damage caused by chemical exposures such as solvents, was tested before and after each session. Peak flow was used as a pre- and postsession measure to determine impairment in lung function. All these measures were recorded pre- and postsession during all booth sessions for each subject.

### Physiologic measures.

To assess physiologic measures of skin temperature, skin conductance, respiratory rate, heart rate, and surface electromyography (EMG), we used used the BIOPAC MP 100 data acquisition system (BIOPAC Systems Inc.) during each booth session. Surface EMG was collected at a rate of 1,000 samples/sec using the Biopac electrodes placed at the upper trapezius muscle. Disposable electrocardiogram electrodes were used to acquire heart rate at a sampling rate of 1,000 samples/sec. The positive electrode was placed on the right wrist, the negative on the left wrist, and the ground electrode on the right ankle. Finger temperature and skin conductance were measured at a rate of 3 sample/sec.

Of the physiologic measures, only skin conductance was a consistent indicator of adaptation and response to challenge substances postadaptation. Results and discussion in this article are therefore restricted to this physiologic measure.

### Skin conductance recording and analysis.

We recorded skin conductance using Ag/AgCl electrodes filled with isotonic electrolyte jelly and attached to the fore and middle fingers of the left hand. Subjects were asked to wash their hands with lukewarm water before the start of each booth session. Skin conductance data acquisition and analysis were conducted using Acknowledge 3.2.4 software (BIOPAC Systems Inc.). The raw data collected were first smoothed using low-pass filter. The readings were compared with the baseline readings from that day.

Skin conductance response has been described in the literature as having two components: phasic and tonic responses (e.g., Lim et al. 1997). Phasic responses may be evoked even by a discrete stimulus such as subtle variations in environment or even thought processes. Tonic skin conductance response is the baseline level of skin conductance in the-absence of any stimulus. This is known to vary with time in the presence of a stimulus depending upon the psychological state of the individual and their autonomic regulation. We considered only the tonic responses while assessing a positive response to challenge substances in our pilot study. Recordings showed variations in the level of conductance because of artifacts or other factors, and the change in amplitude and the length of the tonic response cannot be easily used in a continuous form without arbitrary criteria about where and how the measures will be made. Therefore, we adopted a simple criterion that could be easily reproduced: Tonic responses were considered positive if there was a change in amplitude from the preexposure period (of the session) by at least 0.5 microsiemens (μS) about 20 sec after the introduction of the substance.

### Booth session protocol.

In an orientation visit we discussed details of the study with the subjects and answered questions about the study. After consent, an adaptation period allowed subjects to get used to baseline study protocols, such as cognitive testing (Brown-Peterson test), answering questionnaires, getting used to the booth, and wearing nose plugs.

**Figure 2:**
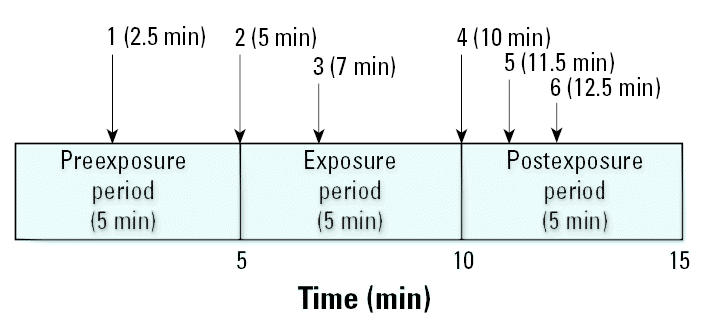
Time line for booth session. Sequence of changes: 1 and 2, opening and closing of side door of booth; 3, exhaust fan on low speed; 4, opening and closing of side door of booth; 5, exhaust fan on high speed; 6, exhaust fan off.

Each subject was given up to 10 individual booth sessions with a maximum of four sessions for adaptation to the baseline study protocols. Each booth session consisted of the same set of changes occurring at the same time, which included opening and closing of the side door through which substances could be introduced (2.5, 5, and 10 min), exhaust fan going on low speed (7 min), high speed (11.5 min), and then being turned off (12.5 min) (Figure 2). The stability in readings was judged only by the stability of tonic skin conductance responses (see above for criteria) and in reduction of symptomatic responses (symptom scores) based on the interview with the nurse.

After adaptation, the subjects were blindly challenged to the test substances, clean air, glue, body wash solution, and dryer sheet in a randomized sequence. Subjects received test substances only if an “open and close” door sequence (time, 2.5 min) in the pretest period, also done during the adaptation phase, did not elicit any change in conductance greater than our defined threshold of 0.5 μS approximately 20 sec after the introduction of the substance. The subjects received only one challenge substance (placebo, control, or test substances) in a session. Each subject was retested to at least one substance that they reacted to in a randomized sequence. Even if they did not react to any substance (as in the case of our control subjects), they were still retested on at least one substance to confirm their nonreactivity.

**Figure 3:**
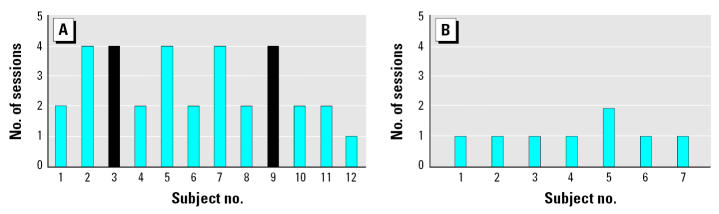
Number of sessions required for each individual to reach stable baseline skin conductance readings (adaptation) among (*A*) cases and (*B*) controls. Subjects 3 and 9 (black bars) did not adapt after four sessions.

Of the 12 subjects with ES, two did not adapt to the baseline protocols and were excluded from further study. All seven controls completed the adaptation phase and were able to participate in the next phase of the study (Figure 3).

This pilot study was single blinded. Subjects were not aware of what substance was introduced and could not smell the substance because of nose plugs. The nurse monitoring subjects was not aware of what was being introduced into the booth. The researcher introducing the substance also monitored data recording and analyzed the results and was separated from the nurse and the subject by a partition. The order of administration of the three test substances (glue, body wash lotion, dryer sheet) and a control substance (unscented shampoo) was randomized using a table of random numbers.

### Data analyses.

Heart rate variability was analyzed by the Institute of Heart Math (McCraty et al. 1995) and did not differ between test and control substances or between patients and controls. Respiratory rate showed erratic patterns that seemed to be influenced by presence of the nose plug, and we could not identify any specific patterns.

In addition to skin conductance, symptom scores before and after booth sessions were the only other measure that indicated completion of an adaptation period and responses to challenge period. No other measures are discussed in this article. Data were dichotomized into reaction versus no reaction to simplify presentation and allow the study to be easily reproduced.

We used SAS (version 9.1; SAS Institute Inc., Cary, NC, USA) for the statistical analyses. Fisher’s exact test statistic (two sided) was used to test differences in proportion using an alpha level of 0.05 between cases and controls, and McNemar’s statistic was used for paired data (between placebo and substance among cases and among controls). Because one column in the 2 × 2 table had 0 frequencies, we used a frequency of 0.001 to be able to estimate a *p*-value.

## Results

Table 1 presents the measures that were collected during the booth sessions, and Figure 2 shows the timeline for the different sequences of the preexposure, exposure period, and post-exposure period.

Figure 3 shows skin conductance responses of cases and controls to baseline study protocols. The proportion of cases reacting to the different testing conditions (different sounds) using set criteria described above decreased with the number of sessions and was much higher in cases than in controls. In the ES group, 83% (10 of 12) adapted after four sessions, whereas 17% (2 of 12) did not adapt after four sessions. Most of the controls adapted in a single session (86%, 6 of 7).

**Figure 4:**
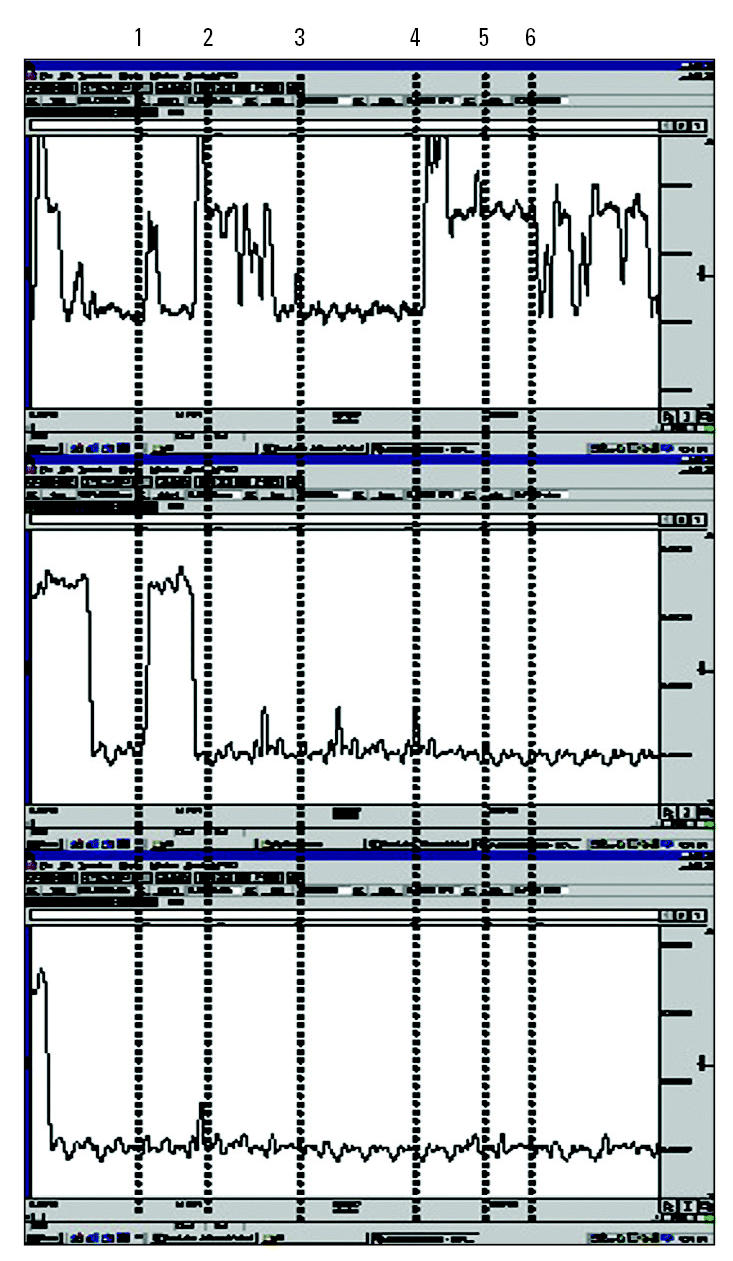
Example of tonic skin conductance responses observed in a case during adaptation, by stimuli and by session. Sequence of changes: 1 and 2, opening and closing of booth side door; 3, exhaust fan on low speed; 4, opening and closing of booth side door; 5, exhaust fan on high speed; 6, exhaust fan off.

**Figure 5:**
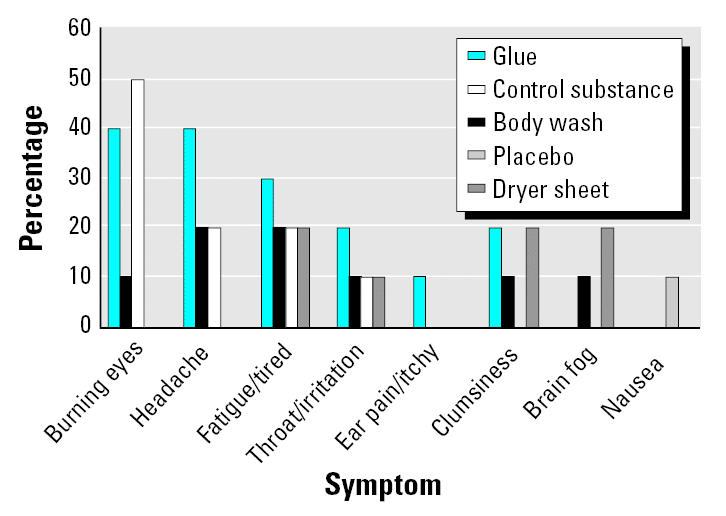
Percentage of individuals with ES presenting responses to challenge substances, by type of symptom and substance (sub).

An example of a tonic skin conductance response during the adaptation period is presented in Figure 4 by stimuli and by session. Although there were variations in skin conductance after the different stimuli in the first and second sessions (first and second window), there were no longer tonic responses in the third session. Figure 5 presents the percentage of individuals with ES having a specific symptomatic response to each of three challenge substances: glue, body wash solution, and dryer sheet. The most common type of reaction was burning eyes and headaches after exposure to the dryer sheet and glue.

**Figure 6:**
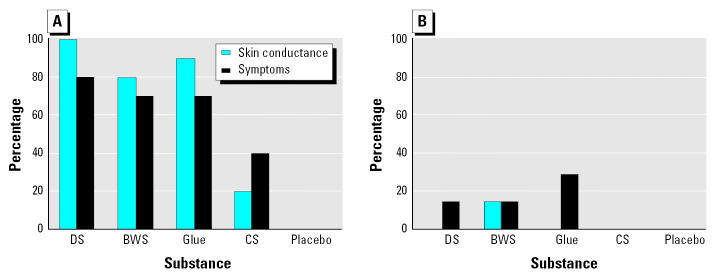
Percentage of (*A*) cases and (*B*) controls presenting skin conductance or any symptom response by test substance. Placebo was clean air. Abbreviations: BWS, body wash solution; CS, control substance; DS, dryer sheet.

The percentages of cases and controls presenting a skin conductance response or any specific symptom to the test substance are shown in Figure 6. The level of response was higher for all test substances in cases than in controls and higher for test substances (glue and dryer sheet) than for control substances (unscented shampoo and clean air) in cases. There was a relatively close match between physiologic and symptomatic responses during exposure to challenge substances. Only one control displayed a response in skin conductance to a test substance, and two controls showed symptom responses to test substances; none showed symptom responses to the control substances.

The most significant difference in skin conductance between cases and controls was for dryer sheets (*p* < 0.0001), followed by glue (*p* = 0.0004) and body wash solution (*p* = 0.02). In cases, comparing skin conductance between substances and control substances, there were similar patterns showing statistical differences between control substance and dryer sheets (*p* = 0.0007), glue (*p* = 0.006), and body wash solution (*p* = 0.02).

For symptomatic responses, there were no statistically significant differences among cases between any substances and the control substances, but there were statistically significant differences between cases and controls for the dryer sheet (*p* = 0.02) and for glue (*p* < 0.05). There were no statistically significant differences among controls.

Table 2 shows symptomatic and skin conductance test–retest results in selected cases and controls by subject and by substance. Table 3 presents all our test–retest data on selected individuals. There was an overall good agreement between test and retest for both symptoms and skin conductance responses (McNemar’s test, *p* = 0.32 and *p* = 0.33, respectively).

## Discussion

The purpose of this pilot study was to shed some light on the experimental conditions, substances, and measures that could lead to better trials in chemically sensitive individuals. There was a clear difference in the time taken by cases to adapt to the experimental conditions, and two cases did not adapt after four sessions; however, more than half adapted after the second session, and six of seven controls adapted in only one session. We have not found any data that emphasize the need for an adaptation period in studies that are measuring physiologic responses outside of the few minutes used to stabilize readings. There is also no mention of the importance of stabilizing the reactions to the experimental conditions such as being observed, changes in temperature and airflow, and sounds such as opening and closing a door to introduce a substance. Although adaptation may not be an important issue in “normal” people, it is certainly an issue in people with ES who are usually in a state of hyper-reactivity. Reactions to test substances need to be differentiated from the effect of the experimental conditions. Otherwise, the experimental “noise” will result in misclassification, reducing statistical significance and resulting in negative findings.

Because we removed most of the noise due to the experimental conditions, the ability of individuals to detect the test substances, compared with placebo and compared with controls, raises several questions. First, EMG, heart rate, respiratory rate, skin temperature, cognition, and contrast sensitivity did not show any consistent patterns of reaction. Therefore, these measures may not be very sensitive or relevant to the pathophysiology of reactions in individuals with ES. Because this was a pilot study, a larger sample size might have shown some more subtle differences, and therefore we cannot totally reject the usefulness of these measures in their ability to discriminate between test and control substances.

Second, differences in skin conductance response bring into question what type of reaction takes place after exposure to a very low-level substance. Skin conductance responses have been widely used in the psychosocial field. Since the work of Edelberg (1972), research has shown that changes in skin conductance (or, conversely, resistance) are affected by the filling of sweat ducts and the number of sweat glands affected. This mechanism, under the autonomic nervous system control, seems cholinergic, regulated by the premotor cortex, the hypothalamus and limbic systems, and the reticular formation. The role of the hypothalamus/limbic system has been previously suggested in the pathophysiology of MCS (Bell et al. 1997). Nevertheless, as Bell and colleagues pointed out, there have been no controlled experiments to determine whether or not sensitization to low-level chemical exposures occurs in MCS patients.

Third, because the hypothalamus/limbic system is closely linked with emotions, is it possible that the perception of chemicals through the eyes (Millqvist et al, 1999) or the upper respiratory system (Shusterman 2002) provoked a rise in anxiety, activating the autonomic nervous system, resulting in a skin conductance response? In future experiments, we will be studying patterns of reactions to see if we can differentiate anxiety responses from the type of response observed in this study.

Fourth, in contrast to a pure anxiety response hypothesis, it could be argued that irritation of these chemosensitive structures (eyes and respiratory tract) could lead to neurogenic inflammation as hypothesized by Meggs (1995) and Bascom et al. (1997). Stimulation of the glossopharyngeal and vagal nerves via the hypopharynx and larynx could result in the type of symptoms described by patients and the observer. Most cases reported burning eyes, eye irritation, headaches, or sleepy or drowsy feelings, which fit with our previous study of symptoms after exposure in chemically sensitive patients (Joffres et al. 2001).

Some of our patients were not able to adapt to baseline protocols, showing erratic responses to the testing conditions. These subjects are therefore in a state of hyperreactivity and should not be included in such experiments. Some cases reacted to the unscented shampoo control substance, but none reacted to clean air. This suggests that clean air should be used as the control substance.

In subjects who adapted to the experimental setup, the most irritant substances (dryer sheets and glue) triggered a physiologic (skin conductance) response accompanied with symptomatic responses in many cases (Figure 6). When we looked at whether symptoms preceded or followed the skin conductance reaction, we found that most symptoms occurred at the same time or followed rather than preceded electrodermal response. Only 20% of subjects had symptoms preceding changes in skin conductance.

Several studies have shown that undetected chemicals can still induce brain activations (Lorig 1994; Sobel et al. 1999). Staudenmayer et al. (1993) used an olfactory masker with the test substances and as a control substance. Therefore, if the olfactory masker is not perceived by the sense of smell but is still able to alter neurophysiology, there should be no detectable differences between test and control substance (olfactory masker) because of the added noise of the olfactory masker. If patients had adapted to the olfactory masker, they might have been able to detect a difference, but the study protocol did not take this problem into account. Lorig (1994) and Sobel et al. (1999) also raised the question of why our controls did not detect the active substances. Perhaps they did not because we considered only tonic skin conductance responses in our study. All but one of our control subjects displayed only phasic responses, whereas almost all the cases showed tonic variations. We will be looking at the importance of phasic changes in our next study. In addition, we did not use measures as sensitive as the electroencephalogram. The pilot study has also helped us identify skin conductance ranges in which a specific type of response, phasic or tonic, is displayed if a stimulus is perceived as a stressor. This will be critical for such studies because these ranges will help us identify what type of response can be considered given a specific range of baseline. We will be confirming this with a larger sample size.

Reaction times varied by substance and were fastest with the dryer sheet, where most of the individuals (8 of 10) reacted in < 200 sec after the introduction of the substance. It is therefore important to allow enough time to observe a response and not introduce other substances that could create experimental noise. Introducing more than one challenge substance in one session, sham exposures, or maskers may not allow isolation of the delayed responses that we observed in a few cases for substances other than the dryer sheet.

Test–retest performed on five cases reacting to glue showed that in four cases the level of reaction was higher in the retest session (similar trends were also observed while retesting on two other test substances), and this fits with Bell et al.’s (1997 (1999) theory regarding sensitization of olfactory-limbic pathways and what Sorg and Newlin (2002) observed in rats, where repeated chemical exposure produced sensitization of the central nervous system circuitry. What was also interesting in the Sorg and Newlin (2002) study was that rats given repeated formaldehyde demonstrated increased fear conditioning to odor paired with footshock, suggesting amplification of neural circuitry guiding fear responding to a conditioned odor cue.

Therefore, whether or not these reactions are triggered by an unconscious anxiety response after the awareness by the nervous system of a situation perceived as threatening can still be argued. We tend to agree with Spurgeon (2002) that the reporting of symptoms may result from a complex set of interactions between aspects of personality, attitudes, culture, and social climate as well as any pathologic changes. Fiedler et al. (2000) noted that reactions to specific substances in individuals do not necessarily elicit physiologic responses. We noted this in a few cases in our study as well. The fact that, in other cases, the physiologic reaction was not followed by any symptomatic response raises the question of whether an anxiety response, recorded at the unconscious level, could still result in such an isolated physiologic reaction.

Symptomatic responses in sensitive individuals and not in controls also correspond to Fiedler et al.’s (2000) results. We are currently investigating whether and how we can differentiate anxiety responses from other types of responses using phasic and tonic responses. Even if we assume an anxiety response, the question remains of whether or not it is an initial, secondary (conditioning), or mixed response to low-level chemical exposure.

There are several limitations to this study. First, because it was a pilot study, the number of cases and controls was relatively small, and results need to be confirmed with a larger study. Then, to ensure a double-blind design, it will be necessary to have two separate individuals, one doing the data analysis and the other in charge of introducing the different substances. In addition, the observer, the nurse, might have been able to pick up the smell of some substances and give unconscious nonverbal cues to the patient inside the booth. We have since installed a video camera that allows remote observation of the patient inside the booth but have not detected any difference in the results due to this change. Although the air flow in the booth was maintained constant and substances were introduced every time in the same manner, it would be essential to measure (with gas chromatography) the actual levels reaching the individuals and ensure that delivery of the substances is constant through methods such as those described by Fiedler et al. (2000).

## Conclusions

In terms of experimental design, this pilot study raised the significance of including an adaptation phase to get stable physiologic measurements and minimize noise in the results. This study also brings up questions regarding the significance of an electrodermal response to low-level chemical substances. Are we observing an unconscious anxiety response, or another type of response such as neurogenic inflammation? Other measures such as functional magnetic resonance imaging would certainly add understanding of the brain regions involved in the reactivity, but this will require unique laboratory conditions. Reproducibility of these results and understanding the pathophysiologic mechanism should be the next priorities.

## Figures and Tables

**Table 1:** Booth sessions measures.

Before	During	After
Interview with nurse	Skin conductance	Interview with nurse
Peak flow	Skin temperature	Peak flow
Contrast sensitivity	EMG	Contrast sensitivity
Brown-Peterson test	Respiratory rate	Brown-Peterson test
Symptoms rating	Electroencephalogram	Symptoms rating
Environment rating	Nurse’s observation	Environment rating

**Table 2:** Symptomatic and skin conductance test–retest results in selected cases and controls by subject and by substance.

Case/control	Test symptom	Retest symptom	Substance	Test skin conductance	Retest skin conductance
Case	1	1	Glue	1	1
Case	1	1	Glue	1	1
Case	1	1	Glue	0 (EMG)	1 (EMG)
Case	0	1	Glue	1 (EMG)	0 (EMG)
Case	1	1	Glue	1	1 (EMG)
Case	0	0	Body wash	1	1
Case	1	1	Body wash	1	0 (EMG)
Case	0	0	Body wash	1	1
Case	1	1	Dryer sheet	1	1 (SKT)
Case	1	1	Dryer sheet	1	1
Case	1	1	Dryer sheet	1	1
Case	0	0	Dryer sheet	1	1 (EMG)
Case	0	0	Placebo	0	0
Case	0	0	Placebo	0	0
Case	0	0	Placebo	0	0
Case	0	1	Control	1	1
Case	1	1	Control	1	0 (EMG)
Case	1	1	Control	0	0
Case	1	1	Control	0	0
Case	0	0	Control	0	0
Control	1	0	Glue	0	0
Control	1	0	Glue	0	0
Control	0	0	Glue	0	0
Control	1	1	Body wash	1	1
Control	0	0	Body wash	0	0
Control	1	0	Dryer sheet	0	0
Control	0	0	Dryer sheet	0	0
Control	0	0	Dryer sheet	0	0
Control	0	0	Control	0	0
Control	0	0	Placebo	0	0
Control	0	0	Placebo	0	0

Abbreviations: 0, no response; 1 positive response; EMG, positive response by EMG; SKT, positive response by skin temperature.

**Table 3:** Examples of test and retest skin and symptomatic responses for selected substances.

		Skin conductance				Symptoms^a^			
		Test		Retest		Test		Retest	
Subject	Test substance	L	A	L	A	Symptom	Scores	Symptom	Scores
1	Glue	210	1.00	600	0.95	Eye irritation	0–7	Ear pain	0–6
						Nausea	1–6		
2	Glue	253	0.86	265	1.38	Eye irritation	0–5	Headache	0–5
						Tired	1–7	Tired	2–7
3	Dryer sheet	300	1.20	500	0.90	Tired	1–8	Tired	0–6
4	Dryer sheet	246	0.90	169	0.98	Headache	3–9	Burning eyes	0–5
5	Body wash	302	0.83	412	0.95	Eye irritation	0–7	Tired	1–6
6 (control)	Body wash	256	1.03	425	1.4	Fatigue	3–9	Fatigue	0–8
7	Control	462	0.95	509	0.86	None		Throat irritation	1–7
								Brain fog	2–8
8	Control	324	1.05	—	—	Brain fog	1–6	Clumsiness	0–7

Abbreviations: —, no data; A, amplitude in microsiemens of tonic skin conductance responses; L, latency in seconds.

a Reported symptoms and symptom severity scores before and after introduction of test substance.
